# Natural and Artificial Photoprotective Agents

**DOI:** 10.3390/molecules26041189

**Published:** 2021-02-23

**Authors:** Diego Sampedro

**Affiliations:** Departamento de Química, Universidad de La Rioja, Centro de Investigación en Síntesis Química (CISQ), Madre de Dios, 53, 26006 Logroño, Spain; diego.sampedro@unirioja.es

Sunlight has a long list of positive effects on living beings. Life on Earth relies on the ability of photosynthetic organisms to make good use of solar energy and convert it into chemical energy. The world as we know it is shaped by the ability of our eyes to respond to incoming light. The metabolism of vitamin D in humans is regulated by solar radiation [1]. These are just some examples of the benefits provided by sunlight. On the other hand, we should also bear in mind that sunlight can also pose a serious danger to life. The deleterious effects of solar radiation on different organisms have been known for a long time. DNA damage, photoinhibition, skin cancer are just some of the risks that different types of organisms have to face [2]. In Nature, evolution has developed different photoprotective mechanisms that allow living beings to cope with the damaging effects of light. The biosynthesis of strongly absorbing compounds and substances able to dissipate light energy work together with antioxidant compounds in order to avoid damage by the Sun. In turn, humans have learnt from these natural photoprotective strategies to design and prepare artificial compounds that can minimize the damage of sunlight to our skin [3]. In this Special Issue, some of the most recent advances in the study of naturally occurring sunscreens and the development of artificial compounds for photoprotection are presented.

Nature is full of examples of well-designed molecules capable of efficiently protecting organisms from direct sunlight and reactive oxygen species (ROS) formed after irradiation. Melanins are a family of compounds found in human skin related with photoprotection and pigmentation. Assis Oliveira et al. report a computational approach to obtain ^15^N NMR shifts of a subset of these pigments in water. Monomers, dimers and tetramers of eumelanin are considered [4]. BML-111, a commercially available agonist of the ALX/FPR2 receptor, seems a promising drug for the reduction on the effects of UVB light. Martinez et al. report on its use in hairless mice [5]. The use of extracts from natural sources is also a promising way to obtain sustainable sunscreens while adding value to the otherwise useless residues. In this sense, Pan and colleagues report the use of extracts from *Phoebe zhennan* wood, mainly containing alcohols and olefins, and useful for the preparation of UV shielding films [6]. 

Using natural compounds as inspiration, many different artificial photoprotective compounds have been designed and prepared. Specific requirements for selected applications or the need to fulfill quite stringent consumer needs have guided the search for new and improved sunscreens. Following this trend, Resende and coauthors report the use of new xanthones to protect the skin from photoaging [7].

In this Special Issue, we collect some reviews from the leading researchers in the field. These contributions provide a comprehensive view of some of the most relevant compounds in photoprotection. Giossi et al. explore the biological role of neoxanthin, a pigment found in plants and algae [8]. The review from Abiola and colleagues focuses on the use of ultrafast spectroscopy and computational methods to understand the photoprotective mechanism of some natural compounds [9]. Demmig-Adams and coauthors present a complete review on the biological roles and properties of zeaxanthin and lutein, carotenoids found in photosynthetic organisms [10]. The use of melanin-related compounds for their use as UV filters and ROS scavengers is reviewed by Solano [11]. Sunscreens could also be used to control the way photochemical reactions take place by filtering specific wavelengths. This concept is developed by Eigvi and Lemcoff in their review [12].

The contributions to this Special Issue stress the importance of photoprotection and its connections to different scientific fields. We still have a lot to learn from Nature when it comes to the design of efficient sunscreens, but this an effort that we need to face to defend ourselves from the damaging effects of sunlight. 

I would like to thank all the authors who have contributed to this issue and the reviewers who helped in the assessment of the scientific quality and relevance. I would also like to thank the staff members of MDPI for their editorial support. Finally, I thank the MINECO/FEDER (CTQ2017-87372-P) for financial support.
